# Isolated Pancreatic Tuberculosis Mimicking Malignancy in an Immunocompetent Host

**DOI:** 10.1155/2012/501246

**Published:** 2012-04-24

**Authors:** Pooja Raghavan, Dhyan Rajan

**Affiliations:** ^1^Department of Medicine, Mount Carmel Health, Columbus, OH 43222, USA; ^2^Department of Medicine, Nassau University Medical Center, East Meadow, NY 11554, USA

## Abstract

Despite the high prevalence of tuberculosis (TB) worldwide, pancreatic TB is rare. When present, pancreatic TB is frequently associated with miliary TB, often in immunocompromised hosts. Pancreatic TB may present as a pancreatic abscess, acute or chronic pancreatitis, and cystic or solid pancreatic masses. We present a case of an immunocompetent patient who presented with two discrete pancreatic masses and was subsequently diagnosed with isolated pancreatic TB. This case suggests that clinicians should have a heightened suspicion of pancreatic TB when faced with discrete pancreatic lesions, especially in patients from areas where the infection is endemic. Such recognition may lead to appropriate diagnostic testing, and possible resolution of pancreatic lesions with antituberculin therapy.

## 1. Introduction

Tuberculosis (TB) involving the pancreas is uncommon, especially when present in immunocompetent hosts [1]. Often occurring in the setting of miliary TB or widely disseminated disease; isolated primary pancreatic TB is extremely rare [1, 2]. Pancreatic TB may present as a pancreatic abscess, acute or chronic pancreatitis, and cystic or solid pancreatic masses [1, 2]. We present a case of isolated pancreatic TB presenting as two discrete pancreatic masses in an immunocompetent host. 

## 2. Case Presentation 

A 20-year-old African male, who recently emigrated from Ghana, presented to the medical emergency room with complaints of abdominal pain and nausea for nearly 2 weeks. The patient stated that approximately 2 weeks ago he began noticing dull, intermittent, midepigastric pain which was neither alleviated nor aggravated by any factors. He also admitted having nausea over the past 10 days, and admitted having a 20-pound unintentional weight loss over the past 6 months. He denied any vomiting, hematemesis, melena, fever, chills, night sweats, and cough. The patient stated that he had previously been in good health and denied the use of any medications. He denied the use of illicit drugs, alcohol, and tobacco and had never participated in any sexual activity. Family history was noncontributory, including the absence of malignancy, or gastrointestinal disorders. Prior to his arrival in the United States 4 weeks ago, purified protein derivative (PPD) testing for tuberculosis yielded an induration of less than 1 centimeter (cm). 

**Figure 1:**
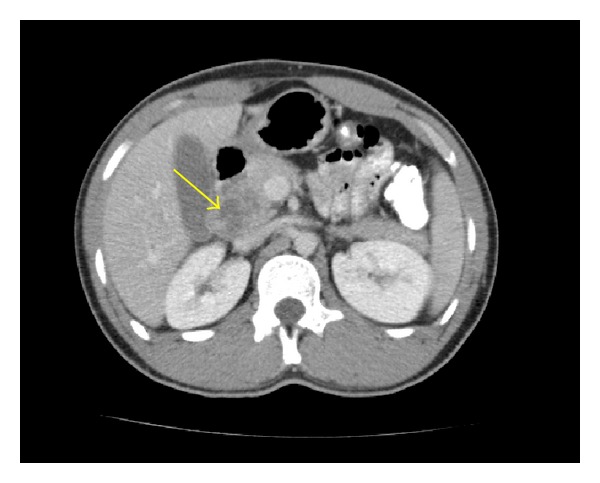
Acomplex cystic mass with thick-walled irregular septations indenting the head of the pancreas (arrow), mimicking the appearance of a cystic neoplasm.

**Figure 2:**
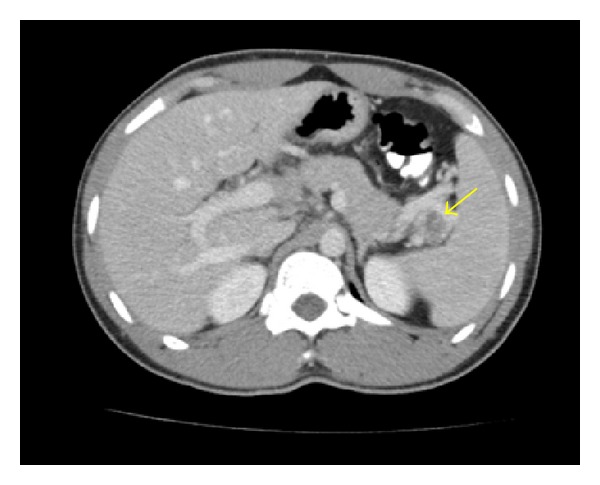
A complex cystic lesion with an internal septation in the splenic porta lying between the tail of the pancreas and the spleen (arrow).

Physical examination was remarkable for mild midepigastric tenderness without guarding or rigidity. Liver transaminases and total bilirubin were within normal range; however there was an increased alkaline phosphatase noted to be 200 U/L. Amylase and lipase levels were also within normal range. Chest X-ray performed was negative for any cardiopulmonary process, and lung fields were noted to be clear. Computed tomography (CT) of the abdomen performed revealed a complex cystic mass with thick-walled irregular septations in head of the pancreas and extending into the porta hepatis, suspicious for a cystic pancreatic neoplasm (Figure 1). Also noted was another complex cystic lesion with an internal septation in the splenic porta lying between the tail of the pancreas and the spleen (Figure 2).

**Figure 3:**
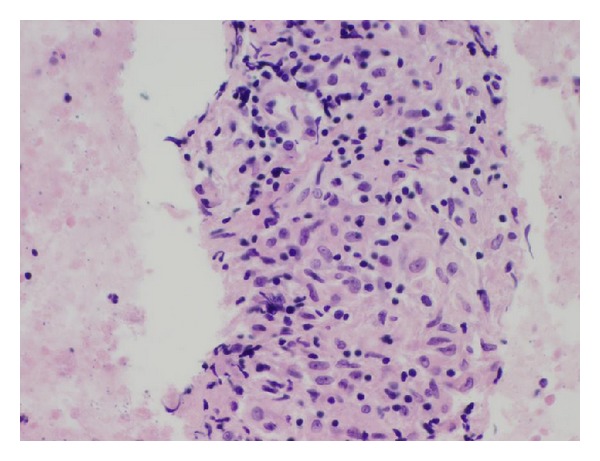
Cell block prepared from the fine needle aspirate of the mass seen in the pancreatic head. Note the appearance of lymphohistiocytic aggregates suggestive of granulomatous inflammation.

Endoscopic ultrasound (EUS) with fine needle aspiration (FNA) of the pancreatic head mass was performed. Sonographically, there was a 3 cm irregular mass within the head of the pancreas extending into the porta hepatis. On-site cytologic and histopathologic evaluation of the biopsy specimen revealed the presence of lymphohistiocytic aggregates suggestive of granulomatous inflammation with no evidence of malignancy (Figure 3). Although smears for acid fast bacilli were negative, a culture of the specimen obtained was remarkable for *Mycobacterium tuberculosis*. 

Given the diagnosis of pancreatic TB, testing for human immunodeficiency virus, a repeat PPD and interferon-*γ* release assay for TB were performed; all yielding negative results. The patient was started on antituberculin therapy with isoniazid, rifampin, pyrazinamide, and ethambutol. The patient reported improvement of symptoms after 3 months of therapy. Repeat imaging of the abdomen performed 3 months after the initiation of antituberculin therapy revealed complete resolution of the two pancreatic masses, and antituberculin therapy was discontinued after a 6-month duration. 

## 3. Discussion

Tuberculosis (TB) is a multisystemic infectious disease caused by various strains of mycobacteria, usually *Mycobacterium tuberculosis*. It is estimated that nearly 9.7 million cases of TB are reported annually; with the highest incidence of infection occurring in Asia, South America, eastern Europe, and most sub-Saharan African countries [3]. Despite a declining incidence of TB in the United States, there were approximately 11,200 new cases of TB reported by the United States Center for Disease Control and Prevention (CDC) in 2010 [4]. Although pulmonary TB is the most common presentation of disease; extrapulmonary TB (EPTB) accounts for nearly 20 percent of all cases of TB in immunocompetent hosts, and nearly 50 percent of all cases of TB in patients with human immunodeficiency virus [5, 6].

By definition, EPTB describes the occurrence of TB at sites other than the lung. The term must not be confused with miliary TB, as this refers to pulmonary involvement with EPTB, and not EPTB in isolation. EPTB can occur in almost any organ system, with the most common sites of infection being the lymph nodes, pleura, genitourinary system, and bone [5, 6]. Abdominal TB is the sixth most common site for EPTB and includes infection anywhere in the gastrointestinal tract, peritoneum, and intra-abdominal organs such as the spleen, liver, and pancreas [6].

Pancreatic TB is rare, with an incidence reported to be less than 4.7 percent worldwide [7]. Isolated pancreatic TB is extremely uncommon, with pancreatic involvement usually occurring in the setting of miliary or widely disseminated TB; often in immunocompromised hosts [1, 5–7]. Infection of the pancreas is thought to occur by direct extension to the organ via lymphatic or hematogenous spread, or by reactivation of previous TB infection [5–7]. Pancreatic TB may present as pancreatic abscesses, acute or chronic pancreatitis, gastrointestinal bleeding, and in rare cases, discrete pancreatic masses mimicking malignancy [5, 8–10].

The clinical presentation of pancreatic TB is often insidious, with nonspecific constitutional symptoms occurring frequently [5]. In a study by Saluja et al., the three most common presenting complaints in patients found to have pancreatic TB were abdominal pain, jaundice, and weight loss [11]. Individuals infected with pancreatic TB may also present with fever, gastrointestinal hemorrhage secondary to splenic vein thrombosis, and anorexia [11]. If pancreatic TB is suspected, preliminary testing such as tuberculin skin testing and an interferon-*γ* release assay for TB may be negative in patients. Sharma et al. suggest that the sensitivity of tuberculin skin testing in patients with abdominal tuberculosis may range from 58 to 100 percent [5]. With the wide-ranging sensitivities of TB screening modalities and an often nonspecific and varied clinical presentation of pancreatic TB; diagnosis of infection relies heavily on radiologic and histopathologic findings. 

Ultrasonography or computed tomography (CT) are often first-line diagnostic modalities in patients presenting with signs of pancreatic pathology [1, 5]. Ultrasonography and CT may reveal both hypodense and hyperechoic lesions, typically found in the head of the pancreas. The findings of these solid or cystic lesions are however nonspecific, as pancreatic adenocarcinomas, cystadenocarcinomas, and pancreatic psuedocysts often have similar appearances [11]. D'cruz et al. further suggest that there is no radiographical difference between cystic neoplasm of the pancreas and pancreatic TB abscess formation, as both present as septated masses with surrounding hypodense lymphadenopathy [12]. As the initial radiographic and clinical presentation of isolated pancreatic TB may mimic malignancy; histologic evaluation of the lesion is essential for diagnosis of pancreatic TB [12, 13]. 

Techniques for pancreatic biopsy include CT or ultrasound-guided percutaneous biopsy, surgical biopsy, or endoscopic ultrasound-(EUS-) guided fine needle aspiration (FNA) [1]. The American Joint Commission on Cancer (AJCC) recommends EUS-FNA as the diagnostic modality of choice in patients with pancreatic masses and has found it to be the most sensitive and specific method for identifying the etiology of pancreatic masses [1]. The presence of on-site cytology is imperative in the diagnosis of pancreatic TB, as the immediate interpretation of the specimen will allow clinicians to request appropriate cultures [1]. The presence of on-site cytology has also been shown to increase the diagnostic yield of FNA by up to 15 percent and may also decrease potential complications by avoiding the need for multiple needle passes once initial diagnostic tissue is procured [1, 14]. Cytologic interpretation of biopsy specimens may reveal the presence of granulomatous inflammation, with the presence of aggregates of epitheloid histiocytes, plasma cells, and lymphocytes [9]. Acid fast bacilli are commonly not seen with FNA. In a study by Farar et al., nearly 40 percent of patients with abdominal TB had staining that was negative for acid fast bacilli [15]. Clinicians should be cognizant of the relatively low yield of FNA specimens to reveal acid fast bacilli and thus culture the specimen for evidence of *Mycobacterium tuberculosis* [9, 15]. Bacterial culture, although requiring a prolonged incubation, has proven to be the most specific diagnostic modality to reveal pancreatic TB [9].

Once the diagnosis of pancreatic TB has been made, standard antituberculin therapy appears to be successful in management of this infection. A minimum of 6 months of antituberculin therapy is often indicated to achieve resolution of pancreatic lesions and alleviation of symptoms. Follow-up CT imaging after treatment may reveal the complete resolution of pancreatic lesions secondary to tuberculosis and may guide clinicians regarding duration of therapy [16]. 

## 4. Conclusion 

Isolated pancreatic TB is extremely rare and may present as discrete pancreatic masses. As the clinical and radiographic presentation may mimic malignancy; clinicians should have heightened suspicion of infectious processes such as TB as a potential etiology of such masses [11]. Furthermore, TB should be considered as a cause of any suspicious pancreatic lesion, especially in patients from areas where the infection is endemic [11]. Clinical awareness of pancreatic TB may guide clinicians to appropriate diagnostic studies and management; which may lead to alleviation of symptoms and possible resolution of pancreatic masses with antituberculin therapy.
